# Implicación del estrés oxidativo en las enfermedades neurodegenerativas y posibles terapias antioxidantes

**DOI:** 10.1515/almed-2022-0022

**Published:** 2022-12-22

**Authors:** Paula Sienes Bailo, Elena Llorente Martín, Pilar Calmarza, Silvia Montolio Breva, Adrián Bravo Gómez, Adela Pozo Giráldez, Joan José Sánchez-Pascuala Callau, Juana Maria Vaquer Santamaría, Anita Dayaldasani Khialani, Concepción Cerdá Micó, Jordi Camps Andreu, Guillermo Sáez Tormo, Isabel Fort Gallifa

**Affiliations:** Servicio de Bioquímica Clínica, Hospital Universitario Miguel Servet, Zaragoza, España; Instituto de Investigación Sanitaria Aragón (IIS Aragón), Zaragoza, España; Comisión de Estrés Oxidativo, Sociedad Española de Medicina de Laboratorio (SEQC-ML), Barcelona, España; Hospital General Universitario Gregorio Marañón, Madrid, España; Centro de Investigación en Red en Enfermedades Cardiovasculares (CIBERCV), Madrid, España; Universidad de Zaragoza, Zaragoza, España; Comisión de Lipoproteínas y Enfermedades Cardiovasculares, SEQC-ML, Barcelona, España; Hospital Universitari de Tarragona Joan XXIII, Tarragona, España; Comisión de Elementos traza, SEQC-ML, Barcelona, España; Servicio de Bioquímica Clínica y Patología Molecular, Hospital Clínico Universitario de Valencia, Valencia, España; Hospital Verge de la Cinta, Tortosa, España; UGD de Laboratorio, Hospital Regional Universitario de Málaga, Málaga, España; Dirección Médica Asistencial, Consorcio Hospital General Universitario de Valencia, Valencia, España; Universitat Rovira i Virgili, Tarragona, España; Hospital Universitari Sant Joan de Reus, Tarragona, España; Institut d’Investigació Sanitària Pere Virgili (IISPV), Tarragona, España; Centre Recerca Biomèdica, Tarragona, España; Unidad de Patología Oxidativa-UPOX-UV, Universidad de Valencia, Valencia, España; Servicio de Análisis Clínicos, Hospital Universitario Doctor Peset, Valencia, España; Laboratori ICS de Tarragona i Terres de l’Ebre, Tarragona, España

**Keywords:** antioxidantes, biomarcadores, enfermedades neurodegenerativas, especies reactivas, estrés oxidativo

## Abstract

**Objetivos:**

El sistema nervioso central es fundamental en el control de la homeostasis y mantenimiento de las funciones fisiológicas del organismo. Sin embargo, sus características bioquímicas hacen que sea especialmente vulnerable al daño oxidativo, lo que compromete su correcto funcionamiento, desencadenando neurodegeneración y muerte neuronal.

**Contenido:**

El estrés oxidativo desempeña un papel importante en la fisiopatología de las enfermedades neurodegenerativas dado que participa en multitud de mecanismos que inducen oxidación de ácidos nucleicos, proteínas y lípidos, contribuyendo con ello, al daño cerebral progresivo. Entre estos mecanismos se encuentran la disfunción mitocondrial, generación excesiva de especies reactivas de oxígeno y nitrógeno, déficit de defensas antioxidantes, oligomerización de proteínas, producción de citoquinas y respuesta inflamatoria, alteración de la barrera hematoencefálica o disfunción del proteasoma. Todas estas disfunciones se han visto implicadas en la patogénesis de diversas enfermedades neurodegenerativas, como la enfermedad de Parkinson, Alzheimer, Huntington o esclerosis lateral amiotrófica.

**Resumen y perspectivas:**

Aunque actualmente no existen tratamientos curativos, las investigaciones se han dirigido a la búsqueda de terapias que permitan reducir el daño secundario al estrés oxidativo y ralentizar la evolución de la enfermedad. Es aquí donde las investigaciones sobre el efecto de las terapias antioxidantes muestran un papel activo.

## Estrès oxidativo y enfermedades neurodegenerativas

**Figura 1:**
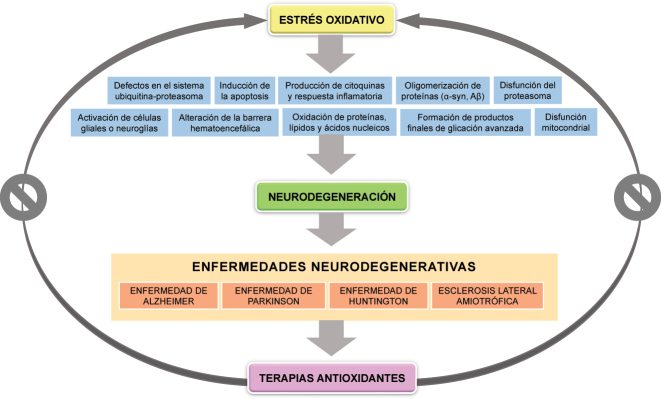
Estrés oxidativo y neurodegeneración. El estrés oxidativo puede participar a través de numerosos mecanismos etiopatogénicos en el proceso de neurodegeneración, característico del grupo de enfermedades conocidas como neurodegenerativas, tales como la enfermedad de Alzheimer, la de Parkinson, la de Huntington y la esclerosis lateral amiotrófica. Por ello, se han desarrollado y ensayado durante años diferentes terapias antioxidantes con el fin de reducir el daño secundario al estrés oxidativo en este grupo de patologías.

Se conoce como neurodegeneración al proceso de degeneración progresiva y muerte neuronal que compromete el correcto funcionamiento del sistema nervioso [1]. La neurodegeneración es el rasgo que caracteriza a un grupo heterogéneo de enfermedades denominadas neurodegenerativas (ENs). En estas enfermedades, el estrés oxidativo (EO), causado por el desequilibrio entre la generación de especies oxidantes y la capacidad de neutralización o reparación de los sistemas endógenos antioxidantes, participa a través de una variedad de mecanismos como oxidación de ácidos nucleicos, proteínas y lípidos; formación de productos finales de glicación avanzada, disfunción mitocondrial, activación de las células gliales, apoptosis, aparición de defectos en el sistema ubiquitina-proteasoma, oligomerización de proteínas como la alfa-sinucleína (α-Syn) o el beta-amiloide (Aβ), producción de citoquinas y respuesta inflamatoria, alteración de la barrera hematoencefálica (BHE) y disfunción del proteasoma [2], [3], [4] (Figura 1).

Entre las especies reactivas de oxígeno (ROS) implicadas en el proceso de neurodegeneración, se encuentran el H_2_O_2_, el radical superóxido (O_2_·^−^) y el radical hidroxilo (·OH), que junto a especies reactivas de nitrógeno (RNS), como el radical óxido nítrico (NO·) y el peroxinitrito (ONOO^−^), poseen un efecto deletéreo sobre las neuronas. Las principales enzimas antioxidantes son la superóxido dismutasa (SOD), la catalasa y la glutatión peroxidasa (GPx). Es importante mencionar que, Los metales de transición, gracias a su potencial redox, desempeñan importantes funciones catalíticas y estructurales en muchos procesos enzimáticos. Sin embargo, su capacidad de aceptar o donar electrones los convierte también en potenciales productores de radicales libres y ROS. En este contexto, destacan las reacciones de Fenton y de Haber-Weiss, en las que se producen radicales ·OH de la reducción del hierro y del H_2_O_2_ [5].

Hay varias particularidades que hacen al cerebro especialmente susceptible al daño producido por EO: (1) alto consumo energético y demanda metabólica, que hacen que desequilibrios mínimos favorezcan la aparición de lesiones tisulares y activen el mecanismo de neuroinflamación; (2) elevado consumo neuronal de oxígeno (20% del total de O_2_ que entra al organismo), asociado a una alta tasa de actividad mitocondrial y relacionado estrechamente con la producción de ROS; (3) alto contenido en lípidos de las membranas neuronales, ricas en ácidos grasos poliinsaturados, altamente susceptibles a la peroxidación lipídica asociada a ROS y metales con actividad redox como cobre, hierro o zinc y (4) las neuronas, a diferencia de otras células, tienen defensas antioxidantes débiles y baja capacidad regenerativa y en ellas los mecanismos de apoptosis están muy restringidos para favorecer su supervivencia [3, 5].

Así, aunque concentraciones bajas-moderadas de ROS y RNS juegan un importante papel en ciertos procesos fisiológicos neuronales (p. ej. vías de señalización, respuesta mitogénica, defensa frente a patógenos, supervivencia celular), su producción excesiva y/o la actividad insuficiente de los mecanismos endógenos neutralizantes, participan en la patogénesis de varias ENs, entre las que destacan la enfermedad de Parkinson, Alzheimer, Huntington, la esclerosis lateral amiotrófica y otras [6, 7]. En esta línea, la identificación en estudios previos de niveles alterados de marcadores séricos de EO en estos pacientes ha puesto de manifiesto el potential de estas biomoléculas en el diagnóstico y pronóstico de este grupo de enfermedades [2, 8, 9].

En esta revisión, se pretende reunir el conocimiento generado sobre la posible implicación del estrés oxidativo en el desarrollo de ciertas ENs. Asimismo, y aunque a día de hoy exista cierta controversia en los resultados publicados, se recogen algunos de los avances y estudios desarrollados en el campo de las terapias antioxidantes dirigidas a la reducción del daño secundario al EO en estas patologías.

### Enfermedad de Alzheimer

La enfermedad de Alzheimer (EA) es la EN más común entre la población anciana. Cursa con deterioro cognitivo y trastornos conductuales, que típicamente se manifiestan por una pérdida de la memoria inmediata y de otras capacidades mentales. Neuropatológicamente, la EA se caracteriza por el depósito de agregados proteicos de Aβ y proteína tau (*τ*), placas amiloides extracelulares de Aβ, ovillos neurofibrilares intracelulares de proteína tau (*τ*) hiperfosforilada y pérdida de las conexiones sinápticas [7].

Diversos estudios sugieren un papel crítico de las ROS y el EO en la patogenia de la EA, debido a su efecto deletéreo sobre las biomoléculas, especialmente sobre las proteínas. Recientemente, mediante el análisis post mórtem de cerebros con EA se ha podido comprobar la presencia de daño oxidativo en enzimas clave del metabolismo energético, proteínas implicadas en la neurotransmisión, enzimas mitocondriales y componentes de la proteasoma [10]. A pesar de que aún existe debate sobre si el aumento de la formación de ROS es causa primaria o consecuencia de la EA, varios estudios han detectado EO severo en estadios tempranos de la enfermedad, incluso antes de que ocurra la acumulación significativa de Aβ [11].

Además, se ha encontrado relación entre el desequilibrio oxidativo inducido por la formación de placas amiloides y niveles elevados de productos de peroxidación lipídica (p. ej. malondialdehído [MDA], 4-hidroxinonenal [HNE]), oxidación de proteínas (p. ej. carbonilo) y de ácidos nucleicos (p. ej. 8-hidroxideoxiguanosina [8OHdG], 8-hidro-xilguanosina), y niveles reducidos de antioxidantes (ácido úrico, albúmina, bilirrubina, licopeno, vitaminas A, C y E y enzimas como la SOD y la catalasa) en los pacientes con EA [6, 9]. El EO también puede agravar la producción y agregación de Aβ y promover la fosforilación de *τ*, lo que podría inducir un círculo vicioso de patogénesis en la EA [12]. Junto a la elevación de ROS, se ha descrito en pacientes con EA un aumento de RNS (p. ej. ONOO^−^), capaces de alterar de forma similar las diferentes biomoléculas y contribuir a la acumulación de Aβ e hiperfosforilación de *τ* [11, 13, 14]. Del mismo modo, se ha observado que el aumento de la expresión de óxido nítrico sintasa (NOS) está directamente asociado con los depósitos de Aβ, lo cual sugiere que los agregados de Aβ podrían inducir a la NOS a producir óxido nítrico, que podría conducir a la formación de 3-nitrotirosina (3-NT) y, con ello, favorecer el desarrollo de EO [15].

Evidencias crecientes sugieren además que la alteración de la distribución de metales como el Zn^2+^, el Fe^3+^ y el Cu^2+^ puede contribuir a la agregación y estrés oxidativo mediado por Aβ y potencialmente a la patología tau [16]. Por último, niveles elevados de ROS estimulan la transcripción de genes proinflamatorios y la liberación de quimiocinas y citoquinas como la IL-1, IL-6 y TNFα, conduciendo a un estado de neuroinflamación y EO crónico que es responsable de la pérdida de neuronas y de la sensibilidad inmunitaria [7, 10].

Sin embargo, como se describe en esta revisión, la distribución desregulada de Cu, Zn y Fe es más importante que la acumulación de tejido a granel y puede contribuir al estrés oxidativo mediado por Aβ y potencialmente a la patología Tau.

Una gran cantidad de evidencia de nuestro grupo y otros en las últimas dos décadas ha indicado que los biometales, a saber, Cu, Zn y Fe, no solo juegan un papel en la agregación de Ab, sino que también facilitan la generación de ROS a través de ligandos Ab:Cu^2+^.

### Enfermedad de Parkinson

La enfermedad de Parkinson (EP) es la segunda EN más común entre la población de más de 60 años, y se caracteriza por síntomas motores como el temblor en reposo o la rigidez. Se produce un deterioro neuronal selectivo de las neuronas dopaminérgicas de la sustancia negra y la formación de inclusiones insolubles de α-Syn conocidas como cuerpos de Lewy [15].

Aunque las vías y mecanismos que conducen a la EP son desconocidos, la identificación de niveles elevados de lípidos, proteínas y ácidos nucléicos oxidados, así como niveles disminuidos de sistemas antioxidantes endógenos como la SOD, la catalasa, el glutatión reducido (GSH) y la glutatión peroxidasa en la sustancia negra de estos pacientes, ha llevado a proponer la participación de las ROS, RNS y el EO en el proceso [5, 17]. Concretamente, la pérdida selectiva de GSH en la sustancia negra y no en otras partes del cerebro, constituye uno de los cambios bioquímicos más tempranos, observados en esta patología [6]. También en otros fluidos corporales como el plasma o el líquido cefalorraquídeo (LCR), varios autores han reportado concentraciones elevadas de MDA y HNE [18]. Asimismo, niveles elevados de varios oligoelementos (Fe^3+^, Mn^2+^, Zn^2+^, Se^2+^, Cu^2+^, Al^3+^) parecen participar en el proceso de neurodegeneración y agravamiento del daño celular que se produce en la sustancia negra por peroxidación lipídica [19].

Incluso se ha propuesto la implicación de modificaciones postraduccionales de α-Syn mediadas por EO, entre las que se incluyen la oxidación, la nitración y la formación de aductos covalentes con HNE, que promueven la oligomerización de esta proteína [10, 20]. Por último, estudios recientes han confirmado que neuronas con mutaciones en proteínas relacionadas con la EP, como α-Syn, DJ-1, LRRK2, PINK1 y parkina, podrían ser más vulnerables al aumento del EO y la disfunción mitocondrial [11, 21].

### Enfermedad de Huntignton

La enfermedad de Huntington (EH) es una EN caracterizada por disfunción motora, demencia y alteración cognitiva progresiva. Está causada por la expansión anómala de tripletes CAG que codifican el aminoácido glutamina, en el exón 1 del gen de la huntingtina (*HTT*). Aunque se ha demostrado que la causa principal de la EH es la toxicidad de la proteína HTT mutada (mHTT), se han identificado otros factores y procesos asociados con la muerte neuronal que caracterizan a esta enfermedad como el plegamiento incorrecto de proteínas, proteólisis anormal, agregación de proteínas, excitotoxicidad y EO, entre otros [8, 20, 22].

El EO tiene un papel relevante y crucial en el curso y evolución de la EH, como marcador de evolución-pronóstico de la enfermedad y de efectividad terapéutica. El desequilibrio oxidativo se produce en esta enfermedad antes de la aparición de los síntomas, lo que indica que el EO y las especies reactivas que se producen, desempeñan un papel clave en la neurodegeneración [23, 24]. El daño oxidativo observado en estos pacientes puede atribuirse a la presencia de depósitos de mHTT que provocan un incremento en los niveles de ROS. Por otro lado, existen evidencias de que la reparación del ADN inducida por daño oxidativo podría conducir a la expansión e inestabilidad de las repeticiones CAG en el gen *HTT* mutado, potenciando así su agregación e induciendo muerte neuronal [8, 20, 25].

Existen varias biomoléculas que desempeñan un papel importante como biomarcadores de EO. En primer lugar, el EO produce un aumento del daño en el ADN (8OHdG), proteínas (grupos carbonilos y nitración de proteínas, 3-NT) y lípidos (MDA, HNE y sustancias reactivas al ácido tiobarbitúrico), entre otros [22, 26]. Además, se produce una disminución en el contenido de GSH y un incremento en las enzimas antioxidantes como GPx, catalasa y SOD [23].

La disfunción mitocondrial se considera uno de los defectos clave en la patogénesis de la EH, lo que se traduce en un aumento de ROS y, por tanto, del daño oxidativo y muerte neuronal. El EO también contribuye a la excitotoxicidad del glutamato, principal neurotransmisor excitante del sistema nervioso central, ya que los radicales libres inhiben la captación del mismo por parte de las células de la glía, aumentando así su concentración extracelular. Esta excitotoxicidad se asocia, a su vez, a un incremento intracelular del ion calcio. La elevación de calcio intracelular se traduce en la activación de la NOS neuronal y la consiguiente liberación de NO que se transformará en ONOO^−^, tras reaccionar con el O_2_·^−^ procedente de la cadena de transporte de electrones. Todo esto induce un desequilibrio entre los sistemas oxidantes y antioxidantes, caracterizado por una producción y liberación excesiva de ROS y RNS y un declive en los sistemas antioxidantes [23].

### Esclerosis lateral amiotrófica

La esclerosis lateral amiotrófica (ELA) se caracteriza por la degeneración progresiva de las neuronas motoras superiores e inferiores de la médula espinal, la corteza y el tronco encefálico. El EO producido por la generación y acumulación de ROS es uno de los principales contribuyentes debido a la alteración del metabolismo energético mitocondrial. Esto está relacionado con la edad, los factores ambientales o la excitotoxicidad mediada por el glutamato, que resulta en una mayor producción de radicales libres [6].

Se han identificado diversos mecanismos implicados en la producción de EO y que dañan las neuronas motoras. En torno al 90% de los casos de ELA son esporádicos, pero el 10% restante resultan de mutaciones en el gen *SOD1*, que codifica la SOD, principal enzima antioxidante neuronal, que cataliza la eliminación de radicales superóxido en una reacción de dismutación y que se traduce en un aumento de la producción de ROS y daño oxidativo. Se han identificado en muestras de sangre, orina, LCR y tejidos de pacientes con ELA varios biomarcadores de EO en proteínas (proteínas carboniladas, 3-NT), productos de peroxidación lipídica y de oxidación de ADN y ARN (8OHdG), demostrando que el EO y las ROS contribuyen de manera significativa al daño cerebral [27, 28]. Las mutaciones en *SOD1* también resultan en disfunción mitocondrial, siendo un componente central de la enfermedad asociada a la fosforilación oxidativa defectuosa, producción de ROS y capacidad de amortiguación del calcio alterada, lo que a su vez, puede potenciar el EO [28], [29], [30].

## Terapias antioxidantes

Debido al papel del EO en el proceso de neurodegeneración, la modulación de la producción de radicales libres o la mitigación de sus efectos perjudiciales se han propuesto como potenciales estrategias terapéuticas para prevenir y controlar algunas ENs [2]. Se ha estudiado el papel neuroprotector de diversos compuestos antioxidantes como posibles terapias para disminuir el daño producido por el EO en estas enfermedades [15]. La mayor limitación de esta estrategia es la ineficacia en la administración de los fármacos, debido a la baja permeabilidad de la BHE, lo que dificulta la introducción de estas moléculas en el ámbito terapéutico de las ENs [3, 5, 21].

### Enfermedad de Alzheimer

La mayoría de los estudios sobre los efectos de la administración de sustancias antioxidantes (vitamina E, vitamina C, ácido lipoico, coenzima Q10 y derivados) han proporcionado información no concluyente, mostrando una disminución del EO, pero sin efectos positivos sobre los aspectos cognitivos o funcionales [6, 21].

Antioxidantes como la coenzima Q10 o su derivado mitoquina mesilato, han mostrado su actividad reduciendo de manera sustancial las placas amiloides en ratones transgénicos para la EA [31, 32]. A pesar de ello, no se ha escalado a estudios clínicos en humanos. Otros tratamientos como la latrepirdine o acetil-L-carnitina (ALCAR) han sido exportados a estudios clínicos. Los resultados no han mostrado la actividad esperada, probablemente debido a que el cambio del paradigma oxidativo no se refleja en una disminución de los cambios ya instaurados en los acúmulos de Aβ o la hiperfosforilación de *τ* [13]. Los efectos de la suplementación con ácidos grasos poliinsaturados (PUFAs) omega-3 en la EA leve han mostrado beneficios en aquellos casos en los que existe un ligero deterioro de la función cerebral. Sin embargo, aunque algunos estudios reflejan cambios en las escalas de la función cognitiva en los casos más graves, no son suficientes para respaldar la suplementación con ácidos grasos omega-3 en el tratamiento de la EA [33].

Los resultados más prometedores se obtuvieron de un estudio que utilizó resveratrol, compuesto capaz de minimizar la agregación de los péptidos Aβ y promover la supervivencia y tolerancia al EO en el sistema nervioso central, actuando como neuroprotector [15, 34].

Actualmente, los inhibidores de la monoaminooxidasa (IMAO) son usados como tratamiento de trastornos neurodegenerativos como la EA, ya que previenen la hiperfosforilación de la proteína tau, el estrés oxidativo y la regulación de los depósitos de Aβ actuando como neuroprotectores. Se han llevado a cabo principalmente investigaciones con IMAOs como la selegilina (IMAO-B) y el ladostigil (IMAO-A y B). La evolución de estos compuestos combinada con la actividad quelante de hierro, podría ser objetivos terapéuticos exitosos en el tratamiento de trastornos neurodegenerativos como la EA [35] (Tabla 1).

**Tabla 1:** Ensayos terapéuticos con agentes/fármacos con propiedades antioxidantes en enfermedades neurodegenerativas.

Terapia antioxidante	Familia	Enfermedad	Modo de acción	Resultados	Referencia
Resveratrol	Polifenol	EA	Disminuye la generación de MDA y nitrito, restaura los niveles de GSH y disminuye la actividad de SOD	Los estudios *in vivo* e *in vitro* muestran que el resveratol puede ayudar al tratamiento de EA, no sólo por sus efectos antioxidantes sino también por sus propiedades neuroprotectoras y efectos antiinflamatorios	Islam et al. [34]
Ácido lipoico	Tiol	EA	Regula al alza los niveles de la enzima glutatión reductasa y reduce el MDA aumentando la actividad antioxidante	Representa un suplemento de potencial significativo en el tratamiento de EA, además de un candidato de efectos positivos sobre la disfunción mitocondrial	Kaur et al. [36]
Acetil-L-carnitina	Derivado acetilado de la L-carnitina	EA	Atenúa significativamente la citotoxicidad, la oxidación de proteínas, la peroxidación de lípidos. Aumenta los niveles de GSH celular	Permiten a los pacientes mantener su rendimiento cognitivo inicial, así como los síntomas conductuales y psicológicos de la demencia durante la duración del tratamiento	Mota et al. [37]
Ácido omega – 3	Ácido graso poliinsaturado	EA	Reduce la peroxidación lipídica y aumenta la actividad de SOD, catalasa y GPx	Muestra beneficios en la aparición de la enfermedad cuando existe un ligero deterioro de la función cerebral	Canhada et al. [33]
Rasagilina, selegilina	IMAO-B	EA	Aumenta la actividad antioxidante y quela hierro	Previenen la hiperfosforilación de la proteína tau y el estrés oxidativo. Regula los depósitos de Aβ. Pueden mejorar la memoria y las capacidades de aprendizaje	Behl et al. [35]
N-acetilcisteína	Tiol	EP	Disminuye la peroxidación lipídica y la actividad de SOD. Aumenta la actividad de GPx y GSH	Parece producir cambios significativos en los niveles de GSH en LCR, aunque sin mejoría inmediata de los síntomas	Niedzielska et al. [6]
Zonisamida	Sulfonamida	EP	Inhibe la liberación de glutamato. Aumento los niveles de 8-OHdG en la orina de pacientes con EP	Muestra eficacia en los síntomas motores de la enfermedad como tratamiento adyuvante de la terapia estándar	Niedzielska et al. [6]
Deferiprona	Quelante de hierro	EP	Capacidad para rescatar células sobrecargadas de hierro al eliminarlo, especialmente de las mitocondrias (los orgánulos más afectados por la acumulación de hierro celular) y, por tanto, reducir la formación de ROS y el consiguiente estrés oxidativo	Los pacientes que comenzaron el tratamiento con deferiprona mostraron un rendimiento motor significativamente mejor a los 6 o 12 meses	Devos et al. [38]
Safranal	Apocarotenoide	EH	Previene la elevación de los niveles de 3-NT y MDA. Disminuye la actividad de SOD, de catalasa y GSH	Efectos beneficiosos sobre la actividad locomotora y el daño oxidativo cerebral en un modelo animal	Fotoohi et al. [39]
Galato de epigalocatequina	Polifenol	EH	Reduce las especies oxidativas reactivas y quela iones metálicos enlazantes	Ayuda a disminuir la acumulación de mHTT	Sebastiani et al. [40]
Riluzol	Benzotiazol	ELA	Bloquea la liberación excesiva de glutamato disminuyendo los niveles de calcio citosólico	Efecto beneficioso leve en la desaceleración de la progresión de la enfermedad y el aumento de la supervivencia	Orrell et al. [41]
			Induce la síntesis de glutatión disminuyendo las ROS		Jaiswal et al. [30]
Edaravona	Pirazolina	ELA	Elimina peróxidos lipídicos y radicales hidroxilo. Reduce los niveles de 3-NT	Puede retrasar la progresión de los trastornos motores funcionales al reducir el estrés oxidativo en pacientes con ELA	Yoshino et al. [42]
					Jaiswal et al. [30]
Curcumina	Compuesto fenólico (diferuloilmetano)	ELA	Atenúa el daño oxidativo y la disfunción mitocondrial	Muestra ligera desaceleración en la progresión de la enfermedad modulando las funciones mitocondriales, mejorando así el estado redox y disminuyendo el daño oxidativo producido	Chico et al. [43]
Vitamina E	Tocoferol	EP	Atenúa los efectos de ROS e inhibe la peroxidación lipídica	No reveló evidencia de ningún efecto beneficioso en la desaceleración del deterioro funcional o en la mejora de las características clínicas de la EP	Shoulson et al. [44]
Coenzima Q10 y derivados (mitoquina mesilato)	Ubiquinona	EA	Disminuye los niveles de MDA y la peroxidación lipídica. Aumenta la actividad de SOD y del complejo I mitocondrial. Reduce el daño oxidativo	Mejora el deterioro cognitivo, el estrés oxidativo, la pérdida sináptica y reduce la acumulación de Aβ, en ratones transgénicos para la EA	McManus et al. [32]
		EP		Juega un papel limitado en el tratamiento de la EP ya que no retarda el deterioro funcional ni proporciona ningún beneficio sintomático para los pacientes con EP	Zhu et al. [45]
		EH		El ensayo no proporciona evidencia de que la Coenzima Q10 retrase la progresión del deterioro funcional en la EH, y estos datos no justifican una recomendación de este compuesto como tratamiento en la EH	McGarry et al. [46]

3-NT, 3-nitrotirosina; 8-OHdG, 8-hidroxideoxiguanosina; Aβ, proteína beta-amiloide; EA, enfermedad de Alzheimer; EH, enfermedad de Huntington; ELA, esclerosis lateral amiotrófica; EP, enfermedad de Parkinson; GPx, glutatión peroxidasa; GSH, glutatión reducido; IMAO-B, inhibidores de la monoaminooxidasa B; LCR, líquido cefalorraquídeo; MDA, malondialdehído; mHTT, proteína HTT mutada; ROS, especies reactivas de oxígeno; SOD, superóxido dismutasa.

Todos estos datos sugieren que el EO tiene un importante papel en la EA. Las terapias antioxidantes muestran como su aplicación induce una disminución de los productos del EO. A pesar de ello, la baja eficacia o el fracaso de los ensayos clínicos puede deberse a que los cambios patológicos producidos en esta enfermedad, como son los depósitos de Aβ y la hiperfosforilación de *τ*, no se modifican eficientemente, incluso cuando se reduce el EO [13].

### Enfermedad de Parkinson

Dada la gran evidencia de la implicación del EO en la enfermedad de Parkinson, se han abordado diferentes estudios para la reducción del daño oxidativo en los pacientes con EP. Desafortunadamente, revisiones y metaanálisis recientes revelan que el suplemento de coenzima Q10, un cofactor esencial involucrado en la fosforilación oxidativa mitocondrial y, por tanto, un potente antioxidante, juega un papel limitado en el tratamiento de la EP ya que no retarda el deterioro funcional ni proporciona ningún beneficio sintomático para los pacientes con EP [31, 45]. De manera similar, la N-acetilcisteína parece producir cambios significativos en los niveles de GSH en LCR, aunque sin mejoría inmediata de los síntomas [6] (Tabla 1).

Debido al aumento de las concentraciones de hierro en la sustancia negra de los pacientes con EP y su capacidad de producir ROS, se ha estudiado el uso de deferiprona, un quelante de hierro, como posible tratamiento. Se ha observado que este fármaco es capaz de eliminar el hierro acumulado y aumentar la actividad de GPx y SOD en LCR reduciendo así la formación de ROS y el consiguiente estrés oxidativo [6, 38]. La vitamina E también se sugirió como una forma de disminuir el EO ya que atenúa los efectos de ROS e inhibe la peroxidación lipídica. Este compuesto es capaz de reducir los síntomas clínicos, pero sin evidencia de ningún efecto beneficioso en el deterioro funcional [6, 44].

Se ha estudiado la administración de zonisamida, un fármaco anticonvulsionante para tratar el temblor en reposo en la EP, ya que parece inhibir el aumento de los niveles de 8-OHdG en la orina de los pacientes con EP, por lo que podría ser de utilidad frente al daño oxidativo [6] (Tabla 1).

La aplicación de terapias antioxidantes de nuevo se fundamenta en la disminución de las ROS y RNS en los pacientes con EP. Si bien los estudios realizados en modelos animales muestran eficacia, las diferencias estructurales respecto al cerebro humano limitan su aplicación, haciendo que las afirmaciones sobre su alcance deban utilizarse todavía con cautela [47].

### Enfermedad de Huntington

Actualmente, no existe tratamiento curativo para la EH y los fármacos usados buscan reducir la corea y los trastornos psiquiátricos. Se han estudiado diversas terapias antioxidantes con el fin de reducir el daño oxidativo y retrasar así la evolución de la enfermedad.

Un estudio en animales reveló que el tratamiento antioxidante con coenzima Q10 parece no tener mucho éxito en los modelos con EH al no prevenir la disminución de la función de la cadena respiratoria cerebral ni aumentar las tasas de supervivencia y la mortalidad. Parece que este compuesto por sí solo no es suficiente para retrasar o detener el progreso de la EH [31].

Un ensayo demostró que el safranal, un compuesto orgánico aislado del azafrán, con actividad antioxidante, previene la disfunción motora en modelos animales de EH. Esto se debe a que este compuesto podría prevenir la elevación de los niveles de nitrito y MDA, así como la disminución de SOD, la actividad de la catalasa y el GSH [39].

El galato de epigalocatequina (EGCG), la catequina más abundante en el té, es un polifenol que se usa en muchos suplementos dietéticos. Las propiedades beneficiosas de este compuesto implican la eliminación de radicales libres, la reducción de especies oxidativas reactivas o la quelación de iones metálicos enlazantes, pueden ayudar a disminuir la acumulación de mHTT y reducir los efectos tóxicos en modelos de EH *in vivo* [40] (Tabla 1).

### Esclerosis lateral amiotrófica

La hipótesis de la participación del EO en la ELA ha generado varios ensayos experimentales. Aunque actualmente no existe tratamiento curativo para esta patología, el único fármaco aprobado por la FDA es el riluzol, antagonista del glutamato, que parece bloquear la liberación excesiva de dicho compuesto disminuyendo los niveles de calcio citosólico con efectos colaterales en la generación de ROS y la función mitocondrial. Existen evidencias que indican que este fármaco disminuye las ROS a través de la inducción de la síntesis de glutatión [28], [29], [30]. Del mismo modo, se ha demostrado que el riluzol presenta un efecto beneficioso discreto en la desaceleración de la progresión de la enfermedad y el aumento de la supervivencia [28, 41].

Se han estudiado varios agentes farmacoterapéuticos con propiedades antioxidantes para retardar la progresión de la ELA, y recientemente se ha demostrado que antioxidantes como la edaravona (aprobado por la FDA en 2017), son eficaces para detener la progresión de la misma durante las primeras etapas, ralentizando el deterioro funcional en algunos pacientes [15, 28, 42]. Se ha observado que este fármaco elimina los radicales de oxígeno, incluidos el radical óxido nítrico y el anión peroxinitrito [30].

Un estudio sobre el tratamiento con curcumina en pacientes con ELA, mostró resultados alentadores que indicaban una ligera desaceleración en la progresión de la enfermedad al modular las funciones mitocondriales mejorando así el estado redox y disminuyendo el daño oxidativo producido [28, 43] (Tabla 1).

## Conclusiones

El estudio del estrés oxidativo en las ENs ha permitido obtener datos muy importantes sobre el mecanismo patogénico subyacente en estas enfermedades, contribuyendo a su mejor conocimiento y a la investigación de biomarcadores para su diagnóstico, pronóstico y seguimiento. El desequilibrio entre la producción de especies reactivas y los sistemas de defensa antioxidante parece ser una condición común en la neurodegeneración. El SNC es muy vulnerable al estrés oxidativo, lo que aumenta la susceptibilidad a la neurodegeneración, desencadenando muerte neuronal y daño cerebral progresivo. Ante la falta de tratamientos curativos para estas enfermedades, la investigación de fármacos con propiedades antioxidantes ha abierto una nueva vía que podría ralentizar los procesos neurodegenerativos.

Bien es cierto que las terapias antioxidantes consiguen disminuir los niveles de EO pero no mediante una acción directa sobre los mecanismos productores del desbalance. Los datos sugieren que los mecanismos implicados en la génesis de ROS y RNS deben ser estudiados en mayor profundidad con el objetivo de conseguir frenar la producción de los mismos y prevenir el desbalance previo a que este esté metabólicamente instaurado. Aunque la mayoría de las estrategias terapéuticas antioxidantes apoyan firmemente que el daño oxidativo contribuye a la neurodegeneración producida en estas enfermedades, las investigaciones en este ámbito son limitadas. Por tanto, futuras investigaciones resultarán de gran ayuda para mejorar la comprensión de la importancia del EO en la fisiopatología de estas enfermedades y su utilidad en la práctica clínica, así como en el desarrollo de posibles tratamientos.

En esta revisión se manifiesta la necesidad de más estudios clínicos para dilucidar el papel de los mecanismos implicados en la génesis de ROS y RNS en el grupo ENs buscando frenar su producción y prevenir el desbalance oxidativo antes de que este se encuentre metabólicamente instaurado. Identificar las rutas metabólicas generadoras de EO permitiría actuar en estos pacientes de un modo más eficaz, sobre el origen del mismo, y no sobre sus productos, pese a que tal y como se ha descrito, la efectividad lograda en la actualidad es todavía muy discreta.
